# Novel Insights into the Mechanisms of Microbial Transcription and Translation

**DOI:** 10.3390/microorganisms11071720

**Published:** 2023-06-30

**Authors:** L. Peter Sarin

**Affiliations:** 1 RNAcious Laboratory, Molecular and Integrative Biosciences Research Programme, Faculty of Biological and Environmental Sciences, University of Helsinki, FI-00014 Helsinki, Finland; peter.sarin@helsinki.fi; Tel.: +358-2941-59533; 2 HiLIFE Helsinki Institute of Life Science, University of Helsinki, FI-00014 Helsinki, Finland

For the better part of the century, microbes have been a treasure trove for deciphering the inner workings of the cell, from early insights into DNA replication and restriction-enzyme-mediated antiviral responses, to unravelling the complexities of metabolic pathways and understanding gene expression and its regulatory mechanisms [1]. The quest for exploring the latter has been significantly boosted by technological strides such as microarrays, high-throughput sequencing, and mass spectrometers, enabling an unprecedented wealth of data on the cell transcriptome, translatome, and proteome [2]. For example, as the publication of this editorial, the National Center for Biotechnology Information (NCBI) featured 539,296 prokaryotic genome sequences. This significantly outnumbers other categories, with viruses coming second with 66,309 genome sequences, and eukaryotes third with 30,380 genome sequences [3]. Nevertheless, does this wealth of genetic information entail a deep understanding of prokaryotic organisms? As is highlighted by the contributions to this Special Issue, the answer is a resounding yes, no, and maybe.

*Escherichia coli* is among the most, if not the most, studied organism, setting a gold standard to which all prokaryotic and many eukaryotic cells are compared. However, even in *E. coli*, transcriptional regulatory networks (TRNs) remain poorly understood, especially regarding their spatial organization. TRNs are crucial for processing and responding to internal and external environmental information in prokaryotic organisms. In their research article, Tian et al. [4] utilize previously published chromatin interaction data of *E. coli* and *Bacillus subtilis* to obtain further insight into the spatial organization within bacterial TRNs. This allowed them to explore various characteristics of bacterial TRNs, including regulation directions, central nodes, hierarchical levels, and network motifs. Their findings revealed that bacterial TRNs exhibit stable spatial organization features under different physiological conditions, which might be associated with biological functions. Importantly, these insights enhance our understanding of the connection between transcriptional regulation and chromosome spatial organization in bacteria [4]. This not only furthers our basic understanding of the cell, but provides key information for enhancing the design of spatial-distance-based gene circuits in synthetic biology applications.

To proceed from gene expression to functional proteins, messenger RNA (mRNA) transcripts need to be translated by the protein synthesis machinery. During prokaryotic translation, initiation can occur through three mechanisms: canonical or Shine–Dalgarno-led initiation, readthrough or 70S scanning initiation, and leaderless initiation. In their review, Leiva and Katz [5] describe the mechanisms of leaderless initiation, characterized by 70S ribosomal particles binding to AUG start codons near the 5′ end of mRNAs. Leaderless mRNAs (lmRNAs) are uncommon in enterobacteria such as *E. coli*, but are prevalent in other bacteria and archaea, where they may constitute a significant proportion of genes. Since lmRNAs lack a 5′ untranslated region and Shine–Dalgarno sequence, their translation regulation differs from Shine–Dalgarno-led translation. Several regulatory mechanisms, including ribosomal RNA processing and changes in the abundance of translation factors, produce global changes in lmRNA translation initiation [5]. Moreover, recent advances in prokaryotic ribosome profiling, such as the first study detailing the archaeal translation machinery [6], will deepen our understanding of the specific regulation of lmRNA translation initiation.

With early bacteriophage studies in the 1950s and 1960s revealing DNA methylation and the existence of restriction enzymes [1], viruses continue to be important tools through which cellular functions can be explored. Viruses are obligate intracellular parasites that entirely rely on the transcription and translation machinery of the host cell for their replication. As outlined in the perspective by Sarin [7], RNA-based regulatory mechanisms have emerged as a focal point for infection studies. Viruses employ a number of these, including the post-transcriptional chemical modification of ribonucleosides, codon usage, and virus-encoded transfer RNAs, as the means to further their replication and evade host immune responses [7]. These RNA-based regulatory mechanisms not only play a crucial role in viral infection, but also provide opportunities for studying host–virus interactions and present potential targets for antiviral strategies. To this end, Pilotto and Werner [8] review the mechanisms by which cellular RNA polymerases (RNAPs) are inhibited, focusing on two viral repressors: RNAP inhibitory protein (RIP) and transcript cleavage factor 4 (TFS4), and their distinct mechanisms of action. RIP blocks the DNA-binding channel and mimics the initiation factor TFB/TFIIB, locking the clamp in a fixed position, and thus impeding RNAP conformational changes that are critical for transcription. TFS4 similarly interacts with RNAP to transcript cleavage factors, interfering with the trigger loop and bridge helix within the active site through occlusion and allosteric mechanisms. These conformational changes are universally conserved; they can be found in both inactive dimers of eukaryotic RNAPI and inhibited RNAP complexes of bacterial and eukaryotic RNA polymerases. Hence, elucidating these inhibitory mechanisms advances RNAP structure–function studies and presents potential targets for antibiotic development [8].

Recent studies have revealed that eukaryotic and prokaryotic viruses alike cause alterations at post-transcriptional ribonucleoside modification (PTM) levels, with specific changes being linked to codon recognition and preferential translation of viral transcripts over host mRNAs [7,9]. In their research article, Lampi et al. [10] investigate the dynamics of the transfer RNA (tRNA) modification landscape during Shewanella phage 1/4 infection in the cold-active marine bacterium, *Shewanella glacialimarina*. The study reveals distinct categories of PTMs based on modification level changes at different infection stages. Furthermore, viral transcripts that are expressed during the middle and late stages of infection, such as the major capsid protein, exhibit a clear preference for the UAC codon, despite it not being prevalent in host transcripts. Interestingly, this temporally coincides with an increase in queuosine modification. Queuosine is exclusively found on tRNAs with GUN anticodons, suggesting a correlation between phage codon usage and tRNA modification [10]. However, further studies are required to ascertain the extent to which PTMs are employed by bacteriophages to control prokaryotic translation.

The articles in this Special Issue provide a timely overview of the multifaceted ways through which microbial transcription and translation are regulated. In particular, they highlight the recent advances in our understanding of RNA-based regulatory mechanisms, an emerging focus area that is only beginning to unravel some of its mysteries.
